# Supplementary material for: Single-Cell Transcriptomics Links Loss of Human Pancreatic β-Cell Identity to ER Stress
Source: Cells. 2021 Dec 19;10(12):3585. doi: 10.3390/cells10123585 (PMC8700697; doi:10.3390/cells10123585)
Supplement: Supplementary file 1 [file cells-10-03585-s001.zip › Supplementary Materials - CELLS-1436310.pdf]

## Supplementary Materials

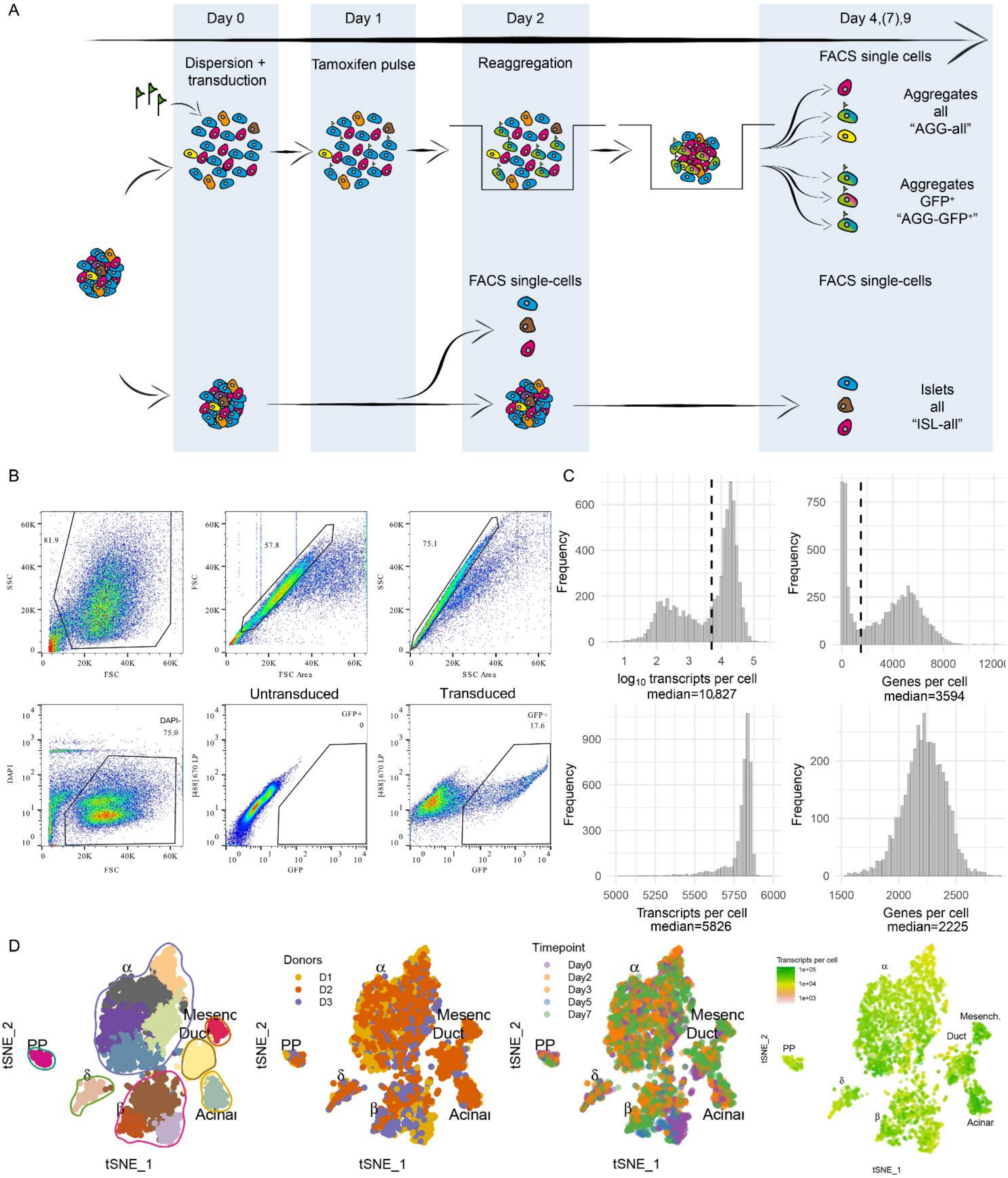


**Figure S1.** Single-cell RNA sequencing to study the adaptive response of human pancreatic β-cells to disruption of islet integrity. (**A**) Scheme of the first experimental approach. Isolated human islets were dispersed into single cells, transduced with a β-cell lineage tracing system and all cells were seeded in microwells (day 0) for reaggregation. Single cells from these aggregates were sorted from the entire live cell population ('AGG-all') or enriched for lineage traced β-cells ('AGG-GFP+') at 2, 5 and 7 days after the start of the reaggregation. Single cells from untransduced intact islets ('ISL-all') were sorted at day 0, 2, 5 and 7. All single cells were sorted and processed for scRNAseq. (**B**) FACS analysis and gate settings to sort viable single cells (cells from Donor 3 are

shown). Cells were distinguished from debris using the forward scatter (FSC) and side scatter (SSC), combining the gates shown in the three plots in the top panel. DAPI exclusion was used to select viable cells (shown in the bottom left plot). The GFP gate was used to enrich for initial β-cells (bottom middle and right). (**C**) scRNAseq data of cells processed using setup 1, as described in A. Transcripts (left) and gene counts (right) per cell pre-filtering (top) and after filtering and normalisation (bottom). Briefly, cells containing less than 5000 transcripts and 1500 genes (indicated by the dashed line) expressed in at least 5% of the cells were discarded. Exclusion of low-quality sequenced cells and downsampling of the transcript counts yielded 4093 cells with a median of 5826 transcripts and 2225 genes per cell. (**D**) t-SNE map highlighting the identified KNN-clusters, which were merged to cell type clusters based on their transcriptional profiles and expression levels of typical markers (shown in Figure 1). t-SNE map highlighting the donor source, showing that cells tend to cluster per donor within the cell types. t-SNE map highlighting the timepoint of sorting for donor 1–3. t-SNE map showing the detected transcripts per cell.

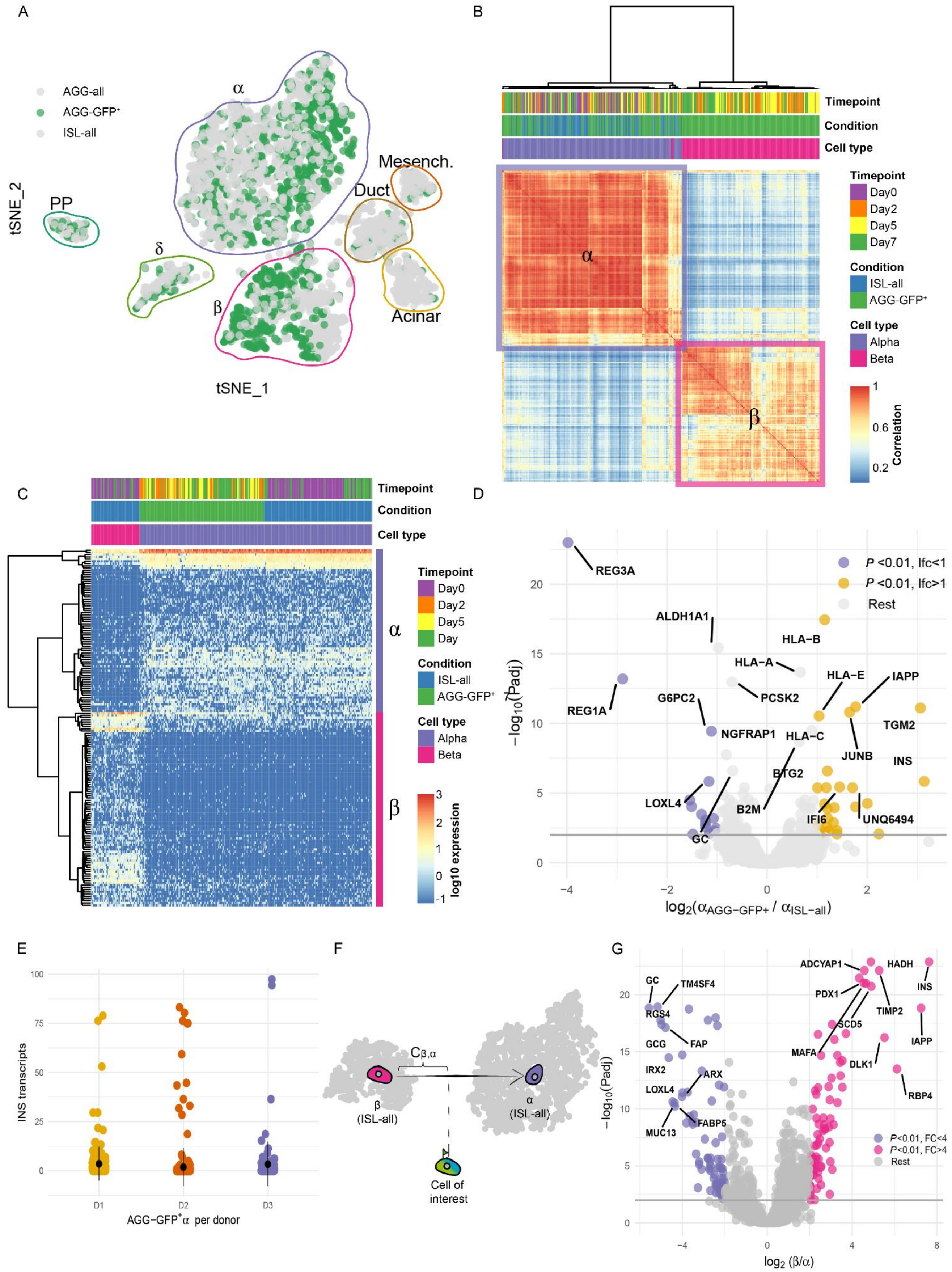


**Figure S2.** Pseudo-temporal ordering of β-cells shows that loss of identity is associated with upregulation of stress markers (1/2). (**A**) tSNE representation of scRNAseq data of cells processed using setup 1 and colour-coded by condition**.** The lineage-traced AGG-GFP+ β-cells are detected in non-β-cell clusters confirming the identity change of

part of the β-cells upon dispersion and reaggregation. (**B**)The lineage traced AGG-GFP+ $\alpha$- and β-cells were compared to canonical $\alpha$-cells sorted from islets (ISL-all condition). The transcriptome-wide similarities represented in the heatmap, in which the intersect of row and column represent the correlation between individual cells, show that AGG-GFP+ $\alpha$-cells cluster with canonical $\alpha$-cells rather than with AGG-GFP+ β-cells, as shown by the hierarchical three on top (donor 3). (**C**) To specifically check the profile of AGG-GFP+ $\alpha$-cells, the expression levels of $\alpha$- and β-cell signature genes are shown in comparison to canonical $\alpha$- and β-cells sorted from islets (ISL-all condition). Genes (rows) are ordered using hierarchical clustering based on their expression. Cells (columns) are grouped based on their cell type and culture condition (donor 3). (**D**) Differentially expressed genes between converted AGG-GFP+ $\alpha$-cells and canonical $\alpha$-cells shown in a volcano plot. Significant genes ($p < 0.01$) upregulated in AGG-GFP+ $\alpha$-cells with log2FC > 1 are coloured in yellow and downregulated with log2FC < 1 in purple. The top 20 genes ($p < 0.01$) are labelled (donor 3). (**E**) Remnant β-cell-specific insulin expression is detected in converted AGG-GFP+ $\alpha$-cells, annotated with average and standard deviation. (**F**) In order to monitor the conversion process in the scRNAseq data, we devised an algorithm to define a cell identity score, shown by the schematic illustration. The score aims at quantifying the theoretical distance of the projection of AGG-GFP$^+$ cells ($C_{\beta\alpha}$) along the trajectory from the average canonical β-cell to the average canonical $\alpha$-cell, sorted from islets (ISL-all). This score allows the pseudo-temporal ordering of the GFP+ labelled cells sorted from aggregates. (**G**) To calculate a cell's identity score, the expression of β- and $\alpha$-specific genes are used. These signature genes define the identity of canonical β- and $\alpha$-cells and are determined by differential expression (with an absolute $\log_2$FC > 2 and adjusted $p$-value < 0.01)) between β- and $\alpha$-cells sorted from islets (ISL-all) and shown here in the volcano plot (donor 3).

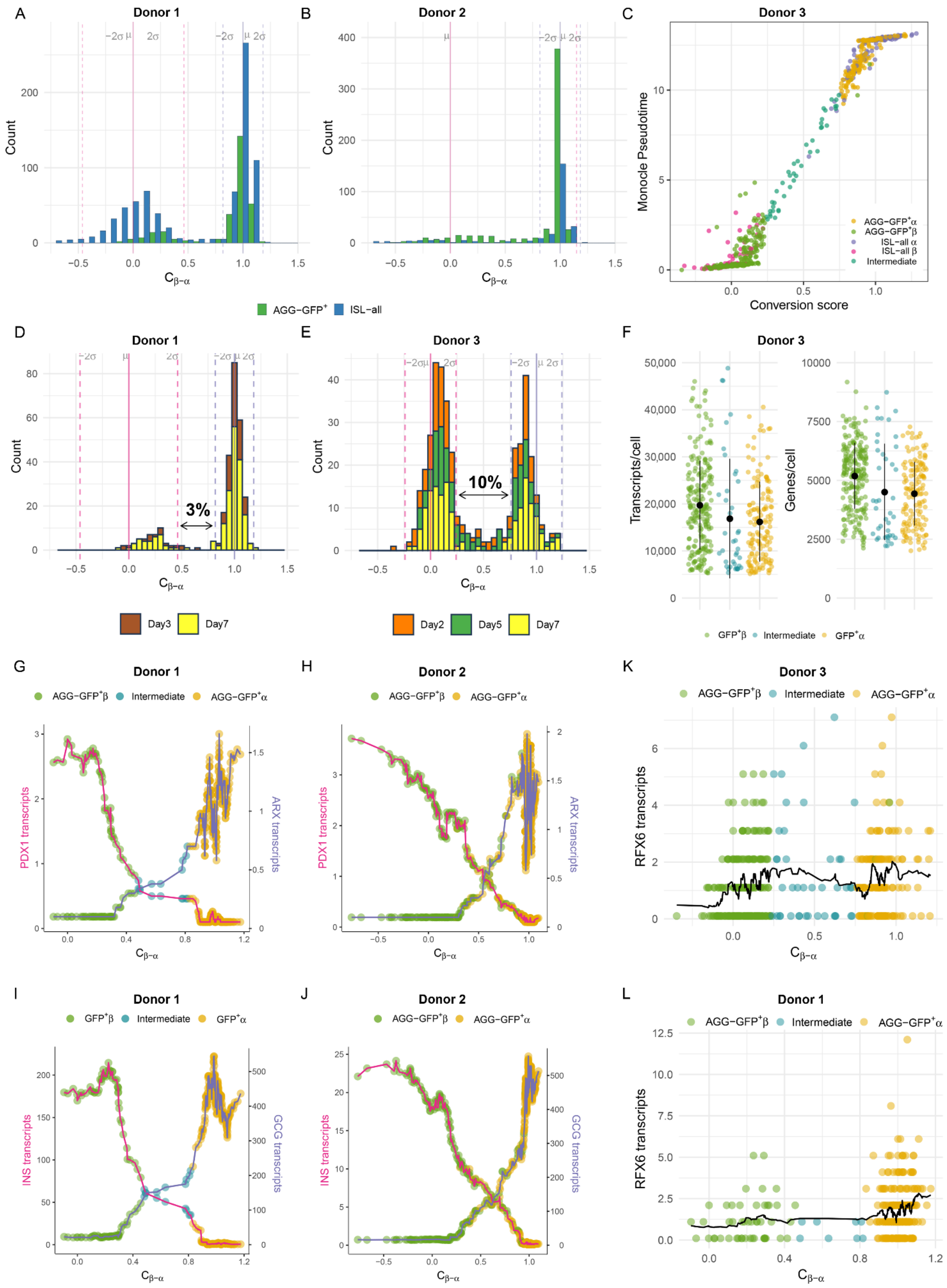

**Figure S3.** Pseudo-temporal ordering of β-cells show that loss of identity is associated with the upregulation of stress markers (2/2). (**A**,**B**) In order to monitor the conversion process in the scRNAseq data, we devised an algorithm to define a cell identity score. Cell identity scores of both canonical (Islet all 'ISL-all'—blue bars) and

Aggregates GFP+ 'AGG-GFP+' (green bars) β- and $\alpha$-cells of donor 1 (**A**) and donor 2 (**B**) are represented. The identity score of canonical β-cells is centred around zero and of canonical $\alpha$-cells is centred around one. The mean (μ) of each canonical cell population and 2 standard deviations from the mean (±2 σ) are indicated by the dashed lines. Because of the very low number of β-cells sorted from islets for donor 2, the estimation of the subpopulation boundaries is hampered and therefore this donor was excluded from further identity score-based analysis. (**C**) The calculated identity score was compared to the most widely used pseudo-temporal analysis algorithm "Monocle". The identity score and Monocle-based pseudo-temporal ordering show a very similar trend with the identity score having more resolution at the edges (donor 3). (**D**,**E**) Identity scores of AGG-GFP+ cells from donor 1 (**C**) and donor 3 (**D**) coloured by time point of sorting post-reaggregation. The histogram shows the distribution and selection of intermediate cells, using the population statics μ and 2σ for each canonical cell population (from ISL-all β and $\alpha$-cells indicated by the dashed lines). (**F**) To exclude that the identity score of intermediate cells is driven by poor cell quality, the RNA sequencing complexity per cell of the intermediate cells was compared to AGG-GFP+ cells. Detected number of transcripts (left) and genes per cell (right) per category indicates similar quality and complexity per cell (donor 3). (**G**,**H**) Concomitant with pseudo-temporal ordering using the identity score, the expression of PDX1 vs. ARX are gradually inverted in AGG-GFP+ cells in donor 1 (**G**) and donor 2 (**H**). The dots represent the moving average of the expression values across identity score, coloured by conversion stage (non-converted β-cells, intermediate and converted $\alpha$-cells). (**I,J**) The expression of insulin vs. glucagon across identity score is gradually inverted in AGG-GFP+ cells in donor 1 (**I**) and donor 2 (**J**). The dotted line represent the moving average of the expression values across the individual cells, coloured by conversion stage (non-converted, intermediate and converted cells). (**K**,**L**) The expression of progenitor marker RFX6 across identity score in donor 3 (**K**) and donor 1 (**L**) is not altered suggesting there is not dedifferentiation to a progenitor stage.

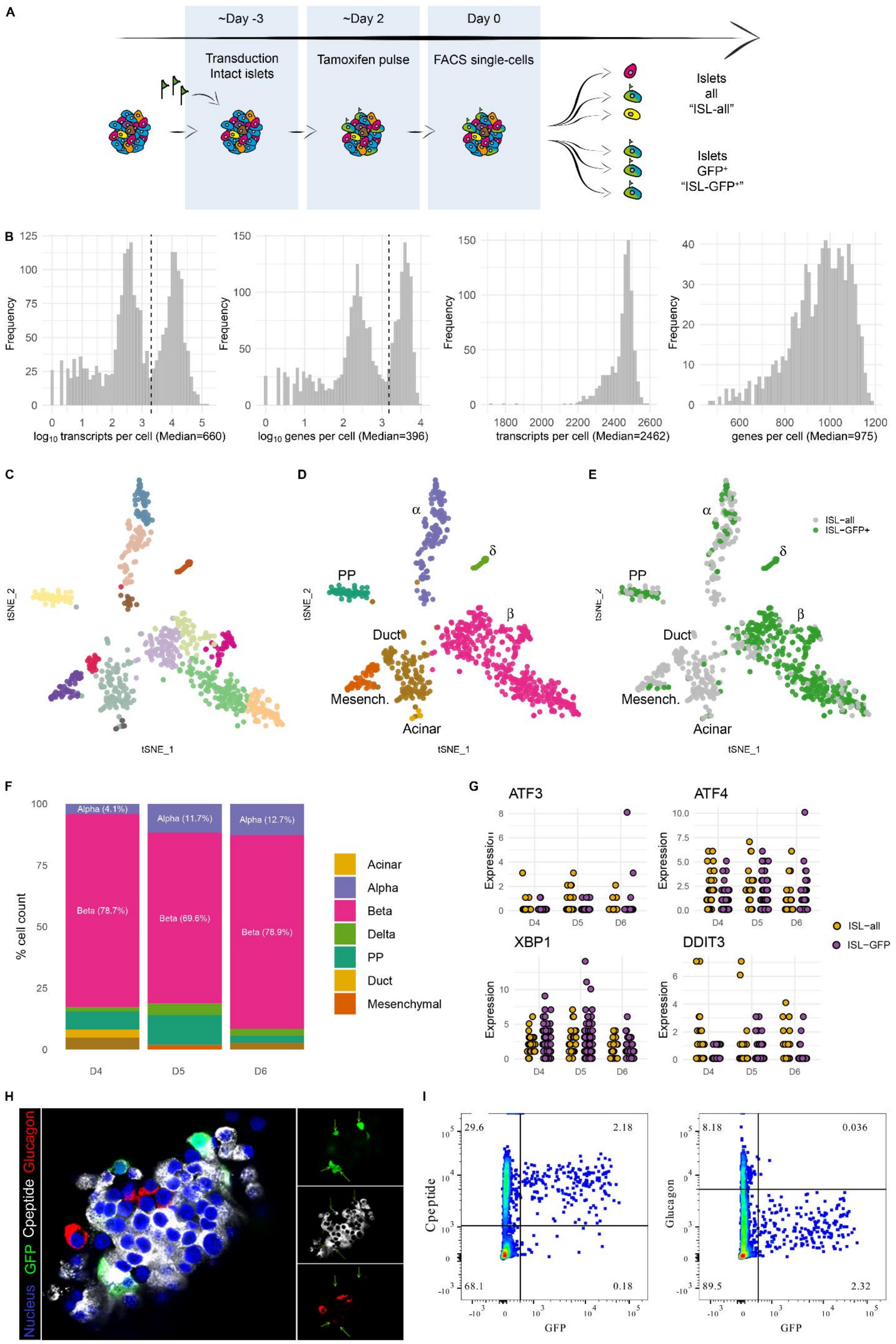
A
~Day -3
~Day 2
Day 0
Transduction
Intact islets
Tamoxifen pulse
FACS single-cells
Islets
all
"ISL-all"
Islets
GFP+
"ISL-GFP+"
B
Frequency
log10 transcripts per cell (Median=660)
log10 genes per cell (Median=396)
transcripts per cell (Median=2462)
genes per cell (Median=975)
C
D
E
tSNE_1
tSNE_2
α
δ
β
PP
Duct
Mesench.
Acinar
ISL-all
ISL-GFP+
F
% cell count
Alpha (4.1%)
Alpha (11.7%)
Alpha (12.7%)
Beta (78.7%)
Beta (69.6%)
Beta (78.9%)
D4
D5
D6
Acinar
Alpha
Beta
Delta
PP
Duct
Mesenchymal
G
ATF3
ATF4
XBP1
DDIT3
Expression
ISL-all
ISL-GFP
H
Nucleus GFP Cpeptide Glucagon
I
29.6
2.18
68.1
0.18
Cpeptide
GFP
8.18
0.036
89.5
2.32
Glucagon

**Figure S4.** Lineage tracing control. (**A**) Second experimental approach used to validate the specificity of the β-cell lineage tracing system. Intact human islets were transduced with the β-cell lineage tracing system (donor 4–6). After 3 to 4 days transduced islets were dispersed and sorted for scRNAseq. (**B**) scRNAseq data of cells processed using setup 2. Transcripts and gene counts (left two panels) per cell pre-filtering and after filtering and normalisation (right two panels). Briefly, cells containing less than 2000 transcripts and 1500 genes (indicated by the dashed lines) expressed in at least 5% of the cells were discarded. Exclusion of low-quality sequenced cells and downsampling of the transcript counts yielded 813 cells with a median of 2462 transcripts and 975 genes per cell. (**C**) Projection of all 813 sequenced single cells for the second experimental setup (donor 4–6) in t-SNE space. Colours show the identified KNN-clusters. (**D**) tSNE-map showing the cell type clusters obtained by merging the KNN-clusters and identified based on their transcriptional profiles and expression of typical marker genes. (**E**) tSNE-map showing the lineage traced ISL-GFP+ β-cells sorted as early as possible after transduction in green. (**F**) Lineage tracing specificity showing the celltypes per GFP+ cells sorted from intact islets (ISL-GFP) per donor. (**G**) scRNAseq data showing no increase in typical ER stress marker expression upon viral transduction of islets (ISL-GFP) in comparison to untransduced islets (ISL-all) (donor 4–6). (**H**) The specificity of the β-cell lineage tracing system was further confirmed at the protein level. Confocal cross-section of an intact islet transduced with the β-cell lineage tracer, illustrates that the β-cell lineage (GFP+ in green) cells are C-peptide positive (white) and glucagon negative (red) (donor 6 is shown, *n* = 2 donors). (**I**) Flow cytometry analysis used to further test the specificity of the β-cell lineage tracing system at the protein level. The majority of the β-cell lineage traced cells (GFP+ on the x-axis) are positive for Cpeptide (left) and negative for glucagon (right) (donor 7).

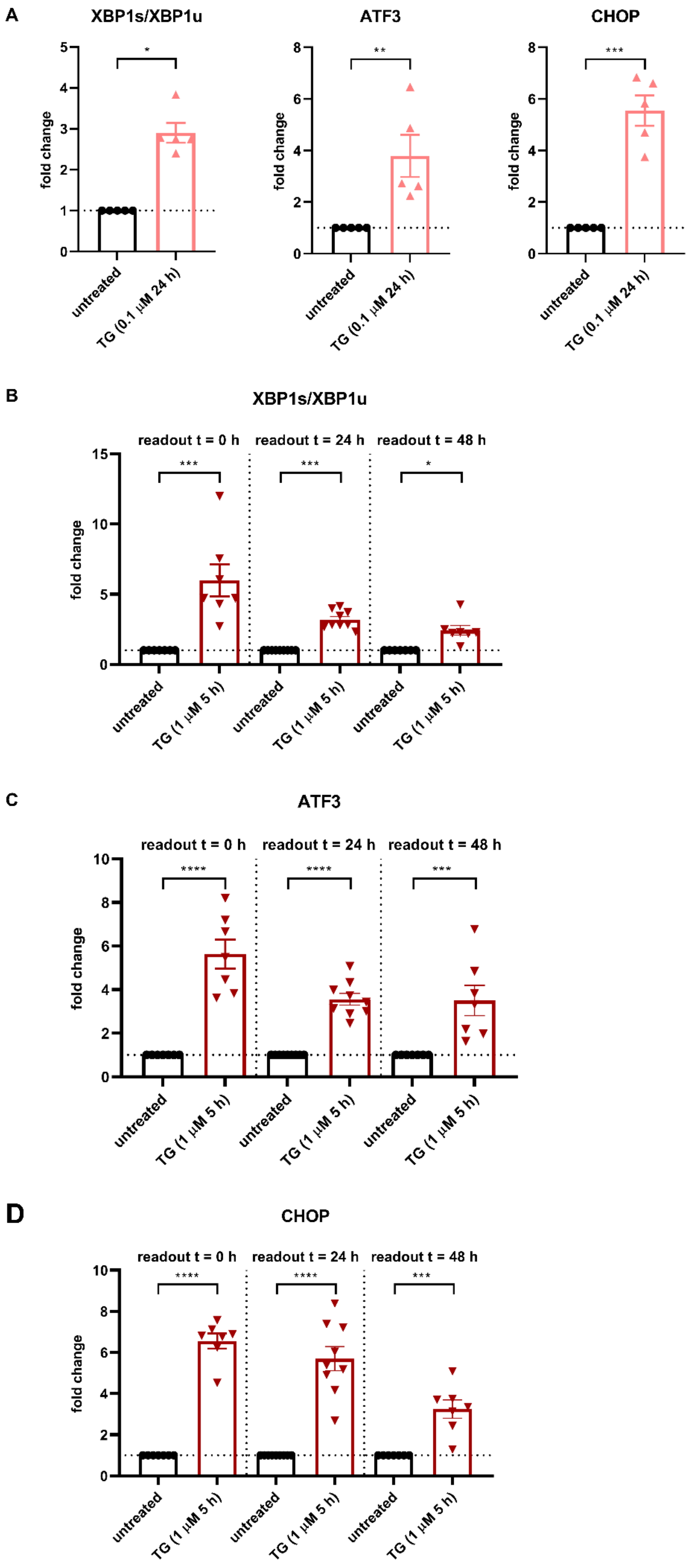
A
XBP1s/XBP1u
ATF3
CHOP
fold change
untreated
TG (0.1 µM 24 h)
*
**
***
B
XBP1s/XBP1u
readout t = 0 h
readout t = 24 h
readout t = 48 h
***
***
*
fold change
untreated
TG (1 µM 5 h)
C
ATF3
readout t = 0 h
readout t = 24 h
readout t = 48 h
****
****
***
fold change
untreated
TG (1 µM 5 h)
D
CHOP
readout t = 0 h
readout t = 24 h
readout t = 48 h
****
****
***
fold change
untreated
TG (1 µM 5 h)

**Figure S5.** Characterisation of the TG model. (**A**) To evaluate the effect of ER stress on β-cell identity, islets were treated with the ER-stress inducing compound thapsigargin (TG) and characterised. Treatment of isolated human islets with 0.1 µM TG for 24 h leads to increased mRNA expression levels of the ER stress marker genes XBP1s/XBP1u, ATF3 and CHOP as measured by qPCR. (**B,C,D**) ER-stress induction in human islets by treatment with 1 µM TG for 5 h leads to increased mRNA expression levels of the ER stress marker genes XBP1s/XBP1u (**B**), ATF3 (**C**) and CHOP (**D**) at 0 h, 24 h and 48 h post treatment, as measured by qPCR. Data are presented as means ± SEM of fold change over non-treated control islets. $n$ = 5–9 donors; each circle represents one donor. * $p < 0.05$, ** $p < 0.01$, *** $p < 0.0005$, **** $p < 0.0001$ vs. non-treated control islets as determined by a paired Student's $t$ test on the dCT values. h = hours, TG = thapsigargin.

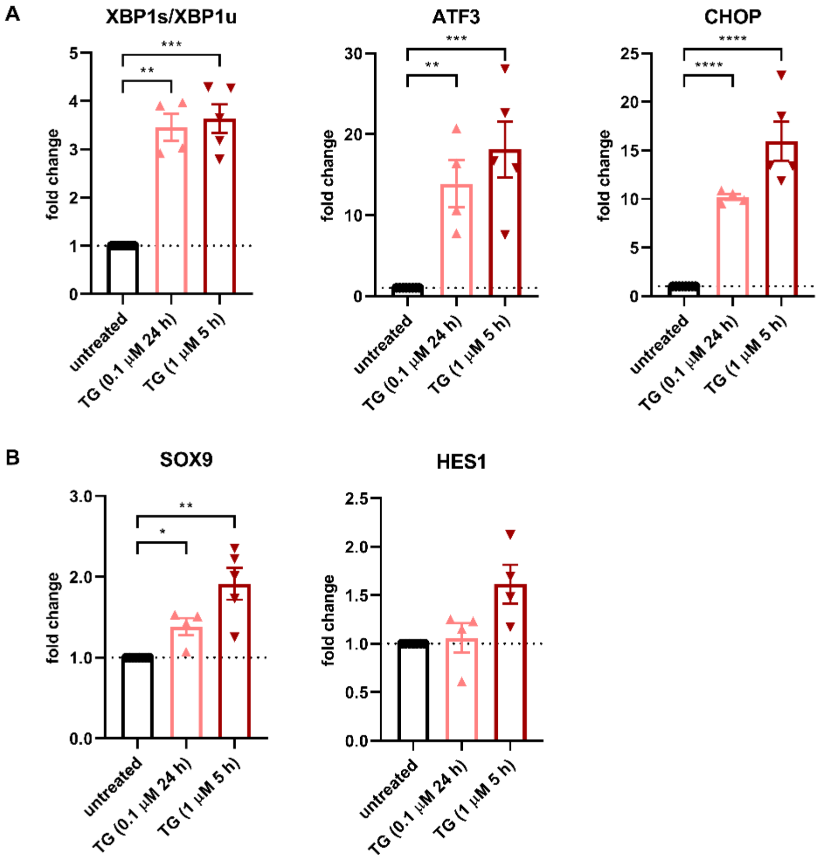


**Figure S6.** Treatment of EndoC-βH1 cells with thapsigargin leads to an upregulation of ER stress markers and endocrine progenitor markers. (**A**) Treatment of EndoC-βH1 cells with 0.1 µM TG for 24 h and 1 µM TG for 5 h leads to increased mRNA expression levels of the ER stress marker genes XBP1s/XBP1u, ATF3 and CHOP as measured by qPCR. (**B**) Thapsigargin-induced ER-stress in EndoC-βH1 cells with 0.1 µM TG for 24 h and 1 µM TG for 5 h leads to increased mRNA expression levels of the endocrine progenitor marker genes SOX9 and HES1 as measured by qPCR. Data are presented as means ± SEM of fold change over non-treated control cells. $n$ = 4–5 batches; each circle represents one batch. * $p < 0.05$, ** $p < 0.01$, *** $p < 0.0005$, **** $p < 0.0001$ vs. non-treated control cells as determined by a paired Student's $t$ test on the dCT values. h = hours, TG = thapsigargin.

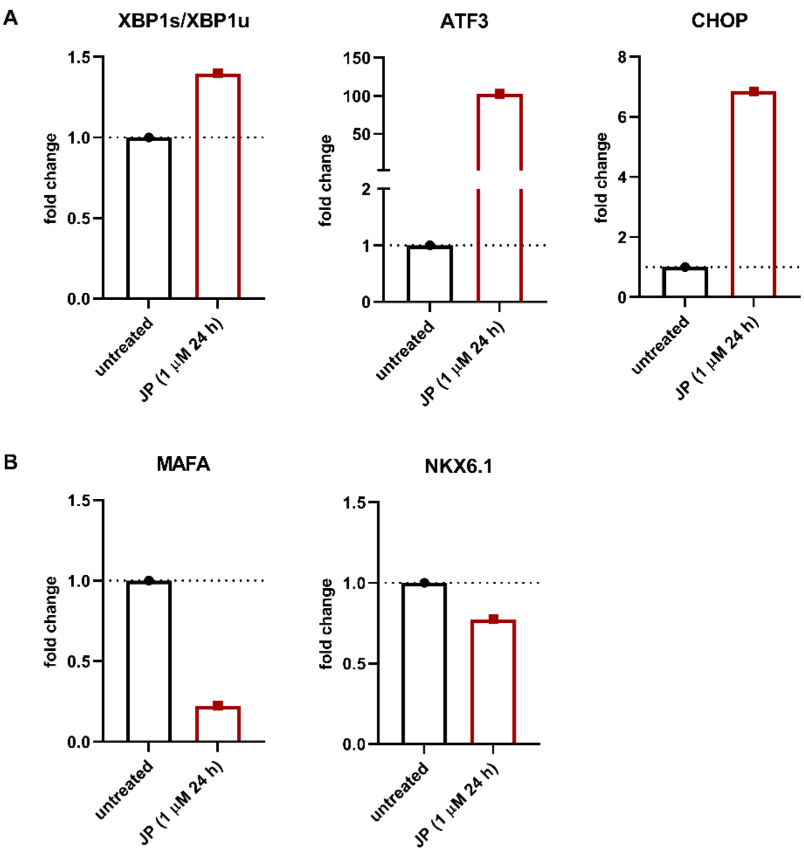


**Figure S7.** Treatment of mature EndoC-βH3 cells with jasplakinolide leads to an upregulation of ER stress markers and a downregulation of β-cell-specific markers. (**A**) Islet integrity disruption is further studied by altering the actin cytoskeleton using Jasplakinolide (JP). Treatment of mature EndoC-βH3 cells with 1 µM JP for 24 h leads to increased mRNA expression levels of the ER stress marker genes XBP1s/XBP1u, ATF3 and CHOP as measured by qPCR. (**B**) Affecting the actin cytoskeleton by treatment of mature EndoC-βH3 cells with 1 µM JP for 24 h leads to decreased mRNA expression levels of the β-cell-specific markers MAFA and NKX6.1 as measured by qPCR. Data are presented as fold change over non-treated control cells. *n* = 1 batch. h = hours, JP = jasplakinolide.

**Table S1.** scRNAseq data composition per donor across cell types and conditions. This table summarizes the number of cells per cell types and conditions for each donor.

**Table S2.** Differential expression analysis of converted AGG-GFP+ α-cells vs. canonical α-cells. This table lists the genes identified as significantly differentially expressed in AGG-GFP+ α-cells vs. ISL-all α-cells for each donor (as separate sheets in the Excel file). Table supplied as Excel file: Table A2.xlsx.

**Table S3.** Signature genes for β-cells. This table lists the signature genes identified by differential expression between canonical β- and α-cells sorted from intact islets (ISL-all) for each donor (as separate sheets in the Excel file). Table supplied as Excel file: Table A3.xlsx.

**Table S4.** Gene expression signature of intermediate cells. This table lists the differentially expressed genes in intermediate cells compared to non-converted β-cells from aggregates on the one hand and to converted AGG-GFP+ α-cells on the other hand for donor 3. Table supplied as Excel file: Table A4.xlsx.

**Table S5.** Effect of dispersion on β-cells. This table lists the differentially expressed genes in β-cells from aggregates compared to canonical β-cells for each donor and time point (as separate sheets in the Excel file). Table supplied as Excel file: Table A5.xlsx.

**Table S6.** Effect of dispersion on α-cells. This table lists the differentially expressed genes in α-cells from aggregates compared to canonical α-cells for each donor and time point (as separate sheets in the Excel file). Table supplied as Excel file: Table A6.xlsx.

**Table S7.** List of human islet donors. This table lists all human islet donors, their characteristics and the estimated purity of the isolated islets used in this study..

**Table S8.** List of qPCR primers. This table lists all qPCR primers and their respective primer sequences used in this study.
